# Straight ladder inclined angle in a field environment: the relationship among actual angle, method of set-up and knowledge

**DOI:** 10.1080/00140139.2015.1115897

**Published:** 2016-03-23

**Authors:** Wen-Ruey Chang, Yueng-Hsiang Huang, Chien-Chi Chang, Christopher Brunette, Nils Fallentin

**Affiliations:** ^a^ Liberty Mutual Research Institute for Safety, Hopkinton, MA, USA; ^b^ Deparrtment of Industrial Engineering and Engineering Management, National Tsing Hua University, Hsinchu, Taiwan, ROC; ^c^ Department of Industrial and Systems Engineering, University of Wisconsin, Madison, WI, USA; ^d^ National Research Centre for the Working Environment, Copenhagen, Denmark

**Keywords:** Ladder set-up, field study, extension ladder, angle measurement

## Abstract

Ladder inclined angle is a critical factor that could lead to a slip at the base of portable straight ladders, a major cause of falls from heights. Despite several methods established to help workers achieve the recommended 75.5° angle for ladder set-up, it remains unclear if these methods are used in practice. This study explored ladder set-up behaviours in a field environment. Professional installers of a company in the cable and other pay TV industry were observed for ladder set-up at their worksites. The results showed that the actual angles of 265 ladder set-ups by 67 participants averaged 67.3° with a standard deviation of 3.22°. Although all the participants had training on recommended ladder set-up methods, only 3 out of 67 participants applied these methods in their daily work and even they failed to achieve the desired 75.5° angle. Therefore, ladder set-up remains problematic in real-world situations.

**Practitioner Summary:** Professional installers of a cable company were observed for portable straight ladder set-up at their worksites. The ladder inclined angle averaged 67.3° with a standard deviation of 3.22°, while the recommended angle is 75.5°. Only a few participants used the methods that they learned during training in their daily work.

## 1. Introduction

Data from the Liberty Mutual Workplace Safety Index (Liberty Mutual Research Institute for Safety 2014) show that the costs for disabling workplace injuries in 2012 due to falls to a lower level in the USA were estimated to be approximately 5.12 billion US dollars or 8.6% of the total cost burden. Ladder incidents continue to be a major safety problem in occupational injuries despite standards and regulations (Occupational Safety and Health Administration 2014; Pliner, Campbell-Kyureghyan, and Beschorner 2014). Ladders are involved in 1 to 2% of all occupational incidents in industrialised countries and roughly 1 out of every 2000 employees has a ladder incident annually (Axelsson and Carter 1995; Häkkinen, Pesonen, and Rajamaki 1988). In the USA, falls from ladders accounted for 20% of work-related fatal falls in 2010 (Bureau of Labor Statistics 2012).

A slip of the ladder at the base has been a common cause of portable straight ladder incidents (Axelsson and Carter 1995; Björnstig and Johnsson 1992; Häkkinen, Pesonen, and Rajamaki 1988; Hsiao et al. 2008; Lombardi et al. 2011). Inappropriate ladder angles or contaminants on the floor surfaces lead to most of the sliding incidents (Björnstig and Johnsson 1992). In 49% of straight ladder incidents, the inclined angle was less than 65°, reported by Axelsson and Carter (1995). Chang et al. (2004), and Chang, Chang, and Matz (2005) reported that the average friction requirement at the ladder base to support human climbing increased by approximately 75% when the inclined angle was reduced from 75° to 65°. If the inclined angle, the acute angle between the ladder rail and the horizontal as viewed from the side, is too small (ladder base is too far from the wall), the base can slide out. If the angle is too large (the base of the ladder is too close to the wall), there is a risk of the ladder tipping over backwards.

The American National Standards Institute (ANSI) recommends 75.5° for the ladder inclined angle (ANSI 2007). Experimental studies in laboratory (controlled) environments have examined the effectiveness of different set-up methods for achieving a 75.5° inclination angle. The ANSI standards promote two different methods for ladder set-up (ANSI 2007).

• The first is the ‘quarter length rule’, also known as the 4 to 1 method, where the base of the ladder should be set away from the wall at 1/4th of the working length of the ladder. The 4 to 1 method was evaluated by Young and Wogalter (2000), and Campbell and Pagano (2014), resulting in average angles of 73.4° ± 5.67° and 70.1° ± 4.87°, respectively.
• The second method, known as the anthropometric or the stand and reach method, is an illustration sticker required by ANSI to be affixed to portable metal ladders. In this method, users are instructed to face the ladder, place their toes against the front of the side rails at the base of the ladder, stand erect and extend their arms straight out, so that the palms of their hands should touch the top of the rung closest to their shoulder level when the ladder is in the correct position (ANSI 2007). The ANSI-recommended stand and reach has been widely evaluated. The angle set-up results associated with this method are 70.55° ± 4.85° by Young and Wogalter (2000), 74.1° ± 3.2° by Knox and Van Bree (2010), 76.2° ± 3.64° by Campbell and Pagano (2014), and an average of 73.5° by Simeonov et al. (2013).
  Several other methods have been proposed for setting up portable straight ladders at the proper angle.
• From 1977 to 1990, ANSI required ladders to be labelled with a tilted backwards ‘L’ such that the ladder was set up properly with the sides of the ‘L’ parallel to the wall and floor (Switalski and Barnett 2003). The backwards L method was evaluated by Young and Wogalter (2000), Knox and Van Bree (2010) and Campbell and Pagano (2014), resulting in angles of 71.8° ± 3.13°, 72.7° ± 2.7° and 71.7° ± 3.36°, respectively.
• Angle feedback devices, such as a plumb bob or bubble level, mounted directly to the ladder indicating correct inclination have been recommended (Goldsmith 1985; Young and Wogalter 2000). Methods based on instruments also have been widely evaluated and results, in general, were closer to the recommended 75.5° angle and highly consistent across participants, with small variations. For the bubble method, Young and Wogalter (2000) and Campbell and Pagano (2014) reported 75.66° ± 0.26° and 75.2° ± 0.34°, respectively, while Simeonov et al. (2013) reported an average angle of 75.8°. The multimodal indicator method evaluated by Simeonov et al. (2013), in which a ladder accessory provides visual and auditory signals as a direct feedback indication of proximity to the recommended ladder angle, resulted in an average angle of 75.4°.
• In addition, a fireman method was evaluated, similar to the standard stand and reach method, in which the participants extended both arms horizontally and held the side rails with both hands, rather than the closest rung (Simeonov et al. 2012). However, there was no significant difference in the set-up angle with these two methods except for the inexperienced participants (*p* = 0.02).
• Finally, the user preference method has been widely evaluated in which the users select the angle at which they feel comfortable climbing the ladder, although achieving the recommended 75.5° angle is not the goal of this method. The results for the user preference method of ladder set-up angle reported in the literature are 66.9° ± 6.1° by Young and Wogalter (2000), 69.1° ± 5.2° by Knox and Van Bree (2010), and averages of 71.9°, 66.3° and 71.3° by Irvine and Vejvoda (1977), Häkkinen, Pesonen, and Rajamaki (1988) and Simeonov et al. (2013), respectively, and were significantly less than those of all other methods reported in the literature. A typical instruction for the user preference method is to tell the participants to set up the ladders the way they would if they were going to climb to the top. Only participants in the studies by Irvine and Vejvoda (1977), and Häkkinen, Pesonen, and Rajamaki (1988) actually climbed the ladders.

In the 75° method evaluated by Young and Wogalter (2000) and Campbell and Pagano (2014), the participants were told to set up the ladder at a 75° angle without any means of angle measurement. This method resulted in angles of 71.8° ± 4.38° by Young and Wogalter (2000) and 72.2° ± 7.13° by Campbell and Pagano (2014). Young and Wogalter (2000) and Simeonov et al. (2013) reported that all of the methods examined in their respective studies resulted in a statistically significant difference in the inclined angle, but Knox and Van Bree (2010), and Campbell and Pagano (2014) reported that only some of the methods they examined resulted in a statistically significant difference.

Ladder set-up has rarely been examined in field environments. In addition to the laboratory study reported by Knox and Van Bree (2010), they also conducted a study of 100 field measurements and reported in the same paper that the mean inclined angle of portable straight ladders was 67.2° with a standard deviation of 4.8°. However, the angles reported in the study were measured afterward and the experimenters did not observe the ladders being set up. The participants indicated that they did not read the label on the ladder about the set-up method every time they set up a ladder. Instead, they relied on what looked right from past experience. In the pool of Knox and Van Bree (2010) field study participants, only 2% were non-professional users of ladders; 77% used ladders on residential construction sites and 21% made commercial use of ladders (e.g. painters). However, it was not clear if the participants in the study received regular training regarding ladder set-up.

Ladder length has been shown to affect the results of the set-up. Irvine and Vejvoda (1977) asked participants to set up four different lengths of ladders the way they thought appropriate and comfortable for carrying out simulated painting tasks. They reported that longer ladders were set up with steeper inclinations than shorter ladders with a statistical significance (*p* < 0.01): 68.70° for a ladder length of 4.88 m, 71.99° for 7.32 m, 73.07° for 9.75 m and 73.97° for 12.19 m. Shorter and retracted ladders were consistently positioned at shallower angles as compared with longer and extended ladders (Simeonov et al. 2013). This is particularly the case for the no-instruction method as well as the stand and reach method, although the latter was not as significant as the former. For the no-instruction method, the average inclined angles were 69.4° and 74.1° for ladder lengths of 2.75 m and 6.41 m, respectively. For the stand and reach method, the average angles were 72.0° and 75.3° for ladder lengths of 2.75 and 6.41 m, respectively. The application of the multimodal indicator resulted in a considerably reduced average range of 74.4° to 76.8°, while the bubble indicator was extremely accurate with an average range of 75.7° to 75.9° (Simeonov et al. 2013). However, Knox and Van Bree (2010), in their study, did not find the relationship between ladder length and inclined angle statistically significant in field observations.

Experience is another factor that has been evaluated. Irvine and Vejvoda (1977) conducted a laboratory study with homeowner and carpenter participants to set up different length ladders, 4.87, 7.32, 9.75 and 12.19 m long, for simulated painting jobs. They reported that the carpenters had non-significantly larger ladder angles than the homeowners (72.88° vs. 70.98°). The results reported by Simeonov et al. (2013) indicated that experience had no statistically significant effect on the ladder inclined angle.

The purpose of this study was to conduct a needs assessment to explore ladder set-up behaviours in field environments. In the current study, multiple methods with field observations and a questionnaire were used to investigate ladder set-up behaviours and outcomes. In contrast to the field study conducted by Knox and Van Bree (2010), the participants in the current study received regular annual training regarding ladder set-up. The participants were observed while they set up ladders at their regular worksites. They then climbed the ladders to perform actual work on a regular workday. At the end of the workday, the participants filled out a questionnaire regarding their knowledge of set-up angle. The objectives of the current study were to explore the ladder set-up methods used, whether participants’ knowledge about ladder set-up gained in the training carried over into the field and the actual inclined angle measured. We speculated that, since this company’s workers received training, the participants’ ladder set-up angles might be closer to the recommended angle. This study can enhance the scientific literature by conducting a needs assessment of ladder set-up in field environments which has been rarely examined.

## 2. Methods

In the current study, set-ups of extension or straight ladders by workers were observed *in situ*, while they performed their daily duties in a field environment. Participants for the study were recruited from employees of a company classified as ‘a cable and other pay TV service’ industry, based on a business classification with a standard industrial class (SIC) code 4841 and the North American Industry Classification System (NAICS) code 515210, the cable television industry. This company provided ladder set-up safety training for new hires and yearly refreshers.

Within this company, participants were recruited from employees in 16 different geographic locations with different safety records. Prior to the visit, the managers at the locations were asked to distribute flyers and make announcements about the study at their regular meetings. Participants were recruited mainly from their weekly meetings with help from the local management teams.

The participants gave written informed consent. The protocol was approved by the New England Institutional Review Board. These participants were followed to various worksites throughout a normal workday from the beginning to the end of the day. They were observed when they set up and used portable straight ladders outdoors. The participants typically used the ladders to get access to higher locations for installation of cable equipment in residential environments. Ladder set-up situations at each worksite were documented with checklists. The experimenters simply observed participants’ activities without interfering in any way with them. In order to reduce a potential bias caused by the observations, this study was presented to potential participants as a general study on workplace conditions. The management was informed that the study concerned working from heights without revealing the focus on the ladder set-up. A debriefing about the actual goal of the study with every participant occurred at the conclusion of data collection for the whole study.

The participants were observed for evidence of whether a particular set-up method for the inclined angle was used and properly applied. In correctly applying the 4 to 1 method, the participants were observed for evidence of judging the working length of the ladder and the distance from the ladder feet to the wall. Actual measurements of distances were not required. For the stand and reach method, the participants would place their toes against the side rails at the base of the ladder, stand erect, extend their arms and place their palm on top of the rung closest to their shoulder level as recommended by the ANSI (2007).

The ladder’s inclined angle was measured by a research team member after the set-up of the ladder had been completed. The inclined angle was measured along one of the rails using a builder’s angle sensor module (Part number 92346, M-D Building Products, Oklahoma City, Oklahoma, USA) with a precision of 0.1°. Any adjustments to environmental obstacles or hazards that would affect the ladder set-up were noted. The working length and working height for each set-up were documented.

A short questionnaire about ladder set-up was filled out by the participants at the end of the working day. Employees’ knowledge of ladder angle, training background and simple demographic information, including age, body weight, body height, tenure, experience with ladder usage, work hours and shift length, were collected. The participants were first asked about the method that they used to set up the ladder and how they learned the method. Then, they were asked if they had received any training about ladder set-up when they were first hired and whether they received any refresher training about the ladder set-up in the last 12 months. At the end, they were asked about the angle that they were taught during their training, if any.

A linear regression analysis was used to explore the relationship between the inclined angle of the ladder and the ladder length. It has been reported in some published studies conducted mostly in laboratory environments that the ladder length would affect the inclined angle. Since this was a field study in which the situations were very different and not controlled, the ladder length used in the analysis was the working length of the ladder which is the distance between both contact points at the top and bottom of the ladder.

Descriptive analyses were used to analyse the results of the training received, and participants’ knowledge of a safe ladder angle. Demographic information was used to identify any factors that could contribute to the ladder inclined angle.

## 3. Results

A total of 84 male employees participated in this study, but two declined to fill out the ladder set-up questionnaire. Depending on the assignments, only 68 participants used portable straight ladders during the periods of observations for a total of 278 observations of straight ladder usages. Data were further screened individually to remove cases where factors existed that could affect the set-up angle such as obstructions on the ground or insufficient ladder lengths for the tasks. The final data pool for ladder set-up contained the results of 265 observations from 67 participants.

Among 67 participants who had a chance to set up straight ladders without obstructions on the ground, only three participants were observed using the stand and reach method in setting up the ladders. One of them set up straight ladders several times during the day with a range of 65.0° to 68.1°. In addition to the training through the company, he reported that he used to work for a roofing company with his father who taught him. Another participant in this category did not use the stand and reach method at the first worksite visited, but started using the method intermittently at the second worksite (four out of nine). However, this participant was observed to perform it properly only once, resulting in an angle of 65.8°. His other three set-ups were performed very quickly and the method used was judged to be incorrect. The third participant, who was observed using the stand and reach method in his sole use of the straight ladder that day, was on his first day working on his own when he participated in this study. The angle of his set-up was 71.1°.

**Figure 1.**
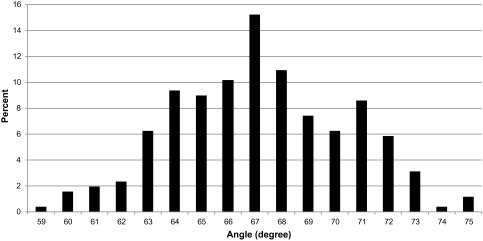
Distribution of the ladder set-up angle (mean 67.3° and standard deviation 3.22°). This distribution was calculated from data of 265 observations from 67 participants.

The overall measured inclined angle had a range from 59.3° to 75.5° with an average of 67.3° and a standard deviation of 3.22°. The distribution of the measured angle is shown in Figure 1.

**Figure 2.**
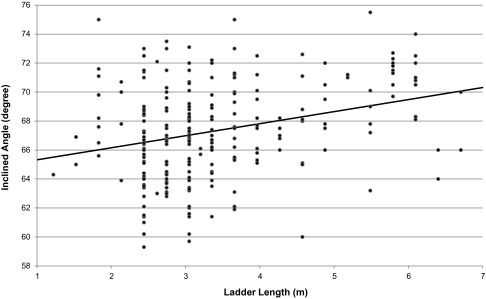
Relationship between the ladder inclined angle and the working length (Pearson correlation coefficient 0.286 with *p* < 0.0001 and *R* ^2^ 0.08).



The Pearson correlation coefficient between the inclined angle and the working length of the ladder was 0.286 (*p* < 0.0001) with an *R* ^2^ of 0.08. The relationship between the measured angle and the working length of the ladder is illustrated in Figure 2. The linear regression equation between the inclined angle and the working length is

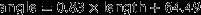



in which the angle is in degrees and the length is in metres.

In the survey questionnaire about ladder set-up that they filled out at the end of the day (*n* = 82), participants were asked about the method that they used to set up the ladders. Among 78 participants out of 82 (95.1%) who answered this question, six (7.7%) checked the user preference, 44 (56.4%) checked 4 to 1, 16 (20.5%) checked stand and reach, 10 (12.8%) checked both 4 to 1 and stand and reach, and two (2.6%) checked more than two methods including 4 to 1, stand and reach and user preference. They were also asked where they learned the methods. Among 82 participants who filled out the questionnaire, 46 (56.1%) wrote company training, 26 (31.7%) put down training without specifying where, six (7.3%) did not give an answer and the other four participants (4.9%) put down firefighter, safety video, experience or previous job. They were asked whether they received any orientation training on setting up ladders when they were hired. Among 82 participants, 79 participants (96.3%) checked yes and three (3.7%) checked no. For the refresher training within the last 12 months on setting up ladders, 76 (92.7%) checked yes, five (6.1%) checked no and one (1.2%) did not answer the question. A majority of the participants received training when they were first hired and also had received refresher training within the last 12 months. While some of them checked no in one of these two questions, none checked no to both questions. They were asked about the set-up method that the training emphasised. Forty-three (52.4%) checked 4 to 1, six (7.3%) checked stand and reach, 24 (29.3%) checked both stand and reach and 4 to 1, one (1.2%) checked user preference, one (1.2%) did not remember and the remaining seven (8.5%) checked different combinations of methods with six (7.3%) of them mentioning 4 to 1 or stand and reach. The participants were also asked about the ladder angle degree that their training recommended. Only two participants answered 75° or 75.5°. Twenty-two simply answered 4 to 1, one answered stand and reach and 20 participants answered values that ranged from 30° to 73°.

Demographics, including age, body weight, body height, tenure, experience with ladder usage, work hours and shift length, were investigated to determine whether they had any effect on ladder inclined angle set-up. The results indicate that the relationship between ladder angle and demographics did not reach a statistically significant level. Since the demographics information included a question about years of experience with straight ladders, the results are in some way consistent with the results reported in the literature showing no significant difference in the ladder angle among experienced and inexperienced users.

## 4. Discussion

This study enhanced the scientific literature by conducting an assessment to explore ladder set-up behaviours in a field environment. Despite several methods that have already been established to help workers achieve the recommended 75.5° angle for ladder set-up as summarised in the Introduction, the results of our study showed that these methods remain rarely used in practice. The actual angles of 265 ladder set-ups by 67 participants averaged 67.3° with a standard deviation of 3.22°, significantly lower than the recommended 75.5°. The reasons why the participants preferred a shallower angle are not clear, but the participants in the field study by Knox and Van Bree (2010) stated that their set-up looked right from past experience. Although all participants had training by the participating company on the recommended ladder set-up methods, and 78 out of 82 participants (95.1%) demonstrated their knowledge in the survey regarding the methods they used to set up the ladders, only three out of 67 participants actually applied these methods in their daily work. There is a significant gap between employees’ knowledge and actual behaviours on ladder set-up.

Nevertheless, the results obtained in the current field experiment are within the ranges of those reported in the literature, most from laboratory studies. The mean angle obtained in the current study, 67.3°, is slightly larger than the 66.9° reported by Young and Wogalter (2000) and 66.3° by Häkkinen, Pesonen, and Rajamaki (1988), and slightly smaller than 69.1° reported by Knox and Van Bree (2010), 71.3° by Simeonov et al. (2013) and 71.9° by Irvine and Vejvoda (1977), all using the user preference method in laboratory environments. The standard deviation of 3.22° obtained in the current study is much smaller than the 6.1° reported by Young and Wogalter (2000) and 5.2° by Knox and Van Bree (2010). By comparison, the Knox and Van Bree (2010) study, based on 100 field measurements of portable straight ladder inclined angles, reported a mean of 67.2° with a standard deviation of 4.8°. Although the mean angles of both studies differed by only 0.1°, in the current study in which the participants received training in ladder set-up, the variation in the angles was smaller than that reported in the field study by Knox and Van Bree (2010) in which the participants did not report training.

The required friction coefficient at the ladder base increased by 73 to 77% on average when the ladder inclined angle was decreased from 75° to 65° (Chang, Chang, and Matz 2005; Chang et al. 2004). The mean angle of the current study was 8.2° lower than the recommended angle of 75.5° for the set-up, so the potential risk of ladder slip-out at the base could increase significantly compared with the risk at 75.5°. In addition to the ladder inclined angle, the chance of a slip-out incident at the base also depends on the climbing height of the user and the available coefficient of friction (ACOF) between the ladder shoes and floor. If the ACOF were sufficiently high, there could be room to allow for a shallower inclined angle without risk of a ladder base slip-out incident, but this is not recommended because the average user could not determine whether the situation allows for a shallower angle, and the options of climbing height and set-up location could be quite restricted in field environments.

Among the results reported in the literature, Irvine and Vejvoda (1977), and Simeonov et al. (2013) reported that the various ladder lengths used in their experiments had a significant effect on the inclined angle. Although the ladder lengths in the current study were not controlled and could be any value, depending on the job requirements, the correlation between the inclined angle and ladder length was also statistically significant. Furthermore, Irvine and Vejvoda (1977) reported an angle of 68.70° for a ladder 4.88 m long vs. 73.97° for 12.19 m with the user preference method. Simeonov et al. (2013) reported average ladder angles of 69.4° and 74.1° for 2.75 and 6.41 m long ladders, respectively, with the no-instruction method, and 72.0° and 75.3° for 2.75 and 6.41 m long ladders, respectively, for the stand and reach method. The results from Irvine and Vejvoda (1977) suggested a rate of increase in ladder length for the angle as 0.72 °/m, while those from Simeonov et al. (2013) suggested a rate of increase in ladder length for the angle as 1.28 and 0.90 °/m for the no-instruction and stand and reach methods, respectively. According to the regression equation shown earlier, a rate of increase in ladder length for the angle at 0.83 °/m for the results obtained in the current experiment is within the range of the data reported in the literature. The results obtained in the current field observation are within the range of results obtained in the laboratories.

It is clearly stated in the training manual of this company that the employees need to maintain the proper ladder climbing angle at a 4:1 ratio: for every 4 feet (1.2 m) of rise, the base of the ladder should be 1 foot (0.3 m) away from the object the ladder is resting against. All the straight ladders have an illustration of the stand and reach method which is a standard requirement for commercially available ladders. In their company training, the installers actually practiced ladder set-up using either the 4 to 1 method or the stand and reach method. In using the 4 to 1 method, they were taught to measure distance by counting the number of rungs and estimating the horizontal distance with their stride. In contrast to most of the previous studies reported in the literature, all the participants in the current study had received training on how to set up ladders and were familiar with the standard set-up methods such as 4 to 1 or stand and reach.

Due to the different heights that needed to be accessed throughout the day, the participants usually carried several ladders on their truck. A long extension ladder, usually 8.53 m long, was typical. They also carried either two additional ladders (a shorter extension ladder and a step ladder) or a combination ladder which could be used as a straight ladder as well as a step ladder. The long ladder was provided by the company, but the rest of the ladders could be provided by the company or owned by the participants. Typically, the step ladders were shorter than the shorter straight ladders. On rare occasions, very short step ladders at the customers’ home were used by the participants out of convenience. For a lower working height, quite often they would just use the step ladder as a straight ladder. This is the reason why some working lengths shown in Figure 2 are very short. The rungs and shoes of a step ladder are angled to be used with the sections opened and spreaders locked. Some of the participants used the step ladder as a straight ladder with the sections closed. This could be dangerous since the rungs and shoes are not designed for this type of usage; the ladder legs could slip out easier and feet could slip off rungs.

Most of the studies about straight ladder set-up have been conducted in laboratory (controlled) environments. Ladder set-up has rarely been examined in field environments. In contrast to the field study reported by Knox and Van Bree (2010), the current study examined the set-up method used as well as the actual outcomes of the set-up (angle), and the knowledge of professional installers of a company in the cable and other pay TV industry. Typical approaches to improve safety are through training that could include knowledge transfer and practices. In the current study, all the participants had received training and a majority of them demonstrated their knowledge about the proper set-up methods through a questionnaire. However, the knowledge gained did not seem to carry over into the field − only three out of 67 participants were observed to use a recommended method to set up their straight ladders. Most of the inclined angles were less than the 75.5° recommended by the ANSI standard. Based on the observations from this experiment, typical behaviours for set-up were simply to place the ladder straight against a wall with no or very little adjustment for shorter ladders and some adjustment with checks for stability and vertical alignment for longer ladders. Most of the time during the set up, the participants looked up toward the upper section of the ladder and they looked down mostly for checking stability. Judgement of the working length of long ladders from the base during ladder set-up could be pretty difficult, so it could be hard to use the 4 to 1 method for the set-up unless they spent time checking for distances.

Even those who properly applied the method of stand and reach achieved inclined angles of 65.0° to 71.1°, which were still much less than the ANSI-recommended 75.5°. Based on the anthropometric data available in the literature, Irvine and Vejvoda (1977) reported that 95% of males would be expected to set up ladders angles between 70.7° and 71.7° with the stand and reach method. Campbell and Pagano (2014) actually measured shoulder height and length of arm from the centre of shoulder to the centre of palm of every participant and arrived at an estimated average of 74.2°. Additional discussion on estimates of the stand and reach method using anthropometric data can be found in Knox and Van Bree (2010).

The cable and other pay TV-installing industry is a fast-paced working environment. The workers are paid by the jobs that they do, so there is an incentive to complete each job quickly as reported in a similar situation in the mail delivery environment (Bentley and Haslam 1998). Although participants said they received training on ladder set-up and were aware of proper set-up methods, most of them did not use the methods taught during training in their daily work. We did not investigate the reasons why the participants did not use these methods, although a fast-paced working environment with incentive to finish jobs quickly could be a potential contributor. The results published by Simeonov et al. (2013) indicated that the times needed to set up a ladder with both the bubble indicator and anthropometric methods were longer than the user preference method. Although they reported a multimodal indicator method which needed even less time than the user preference method, it is not clear if the new method that they evaluated would be widely accepted by the ladder users.

There are some limitations of the current study that can potentially be addressed in future studies. First, *R* ^2^ for the regression equation between the inclined angle and ladder length was only 0.08. Although we could only explain 8% of the variance from the ladder length, we wanted to confirm the relationship between the inclined angle and ladder length in the field environment as reported in laboratory studies. Second, this study explored a potential gap between the recommended ladder set-up methods and actual ladder set-up angles in the field. We did not evaluate the specific training programme used, only whether or not we observed carryover of the training into the field. Due to the non-interference structure of the study, we were not able to give the participants feedback on their ladder set-ups or to ask them the reasons why they chose the methods they did. It could be useful to try to identify the reasons why employees do not use the set-up method that they learn during training, in particular, the behavioral aspects of the ladder set-up. In future studies, a specific training programme can be designed, applying the programme to an experimental group and a control group to demonstrate the impact of the training programme. Also, since the data were collected at only one time point/one day from each employee, longitudinal data can be collected on multiple days from the same employees to demonstrate the consistency in employees’ behaviour in setting up the ladders. Because only one company with one training programme participated in the study, caution should be used when generalising the results to other companies or other industries.

## 5. Summary

This study explored ladder set-up behaviours in a field environment since falls from ladders are an important occupational safety problem. Professional installers of a company in the cable and other pay TV industry were observed for ladder set-up at their actual worksite on one of their typical workdays. At the end of the workday, a questionnaire was used to collect information about their knowledge and training of the ladder inclined angle. The results showed that the actual inclined angles of the ladders set-up by the participants was on average 67.3°, with a standard deviation of 3.22°, which is less than the 75.5° recommended by the ANSI standards. The working length of the ladder was a contributor to the inclined angle set-up, but demographic factors were not. All the participants had training regarding ladder set-up methods, yet only a few participants were observed applying these methods in their daily work. Although the company provided training regarding ladder set-up to all employees and the employees demonstrated their knowledge received during the training through responses in the survey, our study showed that ladder set-up remains problematic when measured in real-world field observations. Although we did not observe any ladder base slip-out incidents during the data collection period, this doesn’t mean that we saw a safe use of the ladders. The set-ups were not carried out according to their training. Ladder incidents are rare events for individuals. Because they are rare, users might not realise the danger of using portable straight ladders until they use the ladder under an unrecognised, unsafe situation. Solutions to the problem may be difficult. One path may be to explore improved and easier to use methods to set up ladders, including the incorporation of devices. The best course forward may be behavioural − employers could take steps to improve the company’s safety climate/culture and to motivate and encourage employees to have better safety behaviour. These additional elements can complement regular training.
